# Learning from errors: assessing final year medical students’ reflection on safety improvement, five year cohort study

**DOI:** 10.1186/s12909-018-1173-7

**Published:** 2018-04-02

**Authors:** Vicki Tully, Douglas Murphy, Evridiki Fioratou, Arun Chaudhuri, James Shaw, Peter Davey

**Affiliations:** 1 Medical School, University of Dundee, Dundee, Scotland; 2 NHS Tayside, Dundee, Scotland

**Keywords:** Patient safety, Medical students, Incident review, Reflection, Professionalism

## Abstract

**Background:**

Investigation of real incidents has been consistently identified by expert reviews and student surveys as a potentially valuable teaching resource for medical students. The aim of this study was to adapt a published method to measure resident doctors’ reflection on quality improvement and evaluate this as an assessment tool for medical students.

**Methods:**

The design is a cohort study. Medical students were prepared with a tutorial in team based learning format and an online Managing Incident Review course. The reliability of the modified Mayo Evaluation of Reflection on Improvement tool (mMERIT) was analysed with Generalizability G-theory. Long term sustainability of assessment of incident review with mMERIT was tested over five consecutive years.

**Results:**

A total of 824 students have completed an incident review using 167 incidents from NHS Tayside’s online reporting system. In order to address the academic practice gap students were supervised by Senior Charge Nurses or Consultants on the wards where the incidents had been reported. Inter-rater reliability was considered sufficiently high to have one assessor for each student report. There was no evidence of a gradient in student marks across the academic year. Marks were significantly higher for students who used Section Questions to structure their reports compared with those who did not. In Year 1 of the study 21 (14%) of 153 mMERIT reports were graded as concern. All 21 of these students achieved the required standard on resubmission. Rates of resubmission were lower (3% to 7%) in subsequent years.

**Conclusions:**

We have shown that mMERIT has high reliability with one rater. mMERIT can be used by students as part of a suite of feedback to help supplement their self-assessment on their learning needs and develop insightful practice to drive their development of quality, safety and person centred professional practice. Incident review addresses the need for workplace based learning and use of real life examples of mistakes, which has been identified by previous studies of education about patient safety in medical schools.

## Background

The importance of authentic clinical incidents for learner engagement in patient safety has been recognised by an Inter-professional Study Group [1] a systematic review [2] and surveys of faculty [3] and students [4]. Existing education tends to describe but not explore or explain how practitioners deviate from best practice, which leaves learners unable to analyse the pathways to error. Incident review could enable medical students to look more explicitly at why practice breaks down and the circumstances in which it does so [1].

In 2010 the Medical School at the University of Dundee agreed that a trial could be carried out to enable all final year students to participate in an incident review [5]. All final year students who were not carrying over work from the previous year were invited to undertake an incident review. We prepared students with a tutorial based on the WHO Learning from Errors Patient Safety Workshop [6] and with an online course. All 126 students who were invited to carry out an incident review completed the assignment. Students investigated the incidents in groups of up to six students and we allowed them to submit individual or group reports. Marked differences were found between individual reports from students who had investigated the same incident [5]. From these findings it was recommended that in future students should investigate incidents in groups but submit individual reports. The Medical School agreed that Incident Review should be a core component of Final Year. A structured reflective report was required, which could be assessed and included in the students’ submission for the Final Portfolio Exam. We identified a method to measure resident doctors’ reflections on quality improvement that could be adapted for medical students: the Mayo Evaluation of Reflection on Improvement Tool (MERIT) [7]. MERIT is based on transformative learning theory, which supports the conceptual framework that healthcare professionals must critically reflect on events in practice in order to develop meaningful improvement solutions [7]. In this paper we describe the use of a modified version (mMERIT) for assessment of medical students’ reflection on safety improvement.

## Methods

We adapted MERIT for use by medical students (Table 1). The modifications were intended to enable medical students to reflect on an incident that had already been reported, whereas the original MERIT asked doctors to reflect on an incident that they had reported. We did not consider that these changes had altered the content and structure validity of MERIT. Consequently the aim of the study was to assess the reliability of the modified (mMERIT) tool and demonstrate sustainability. The design was a five year cohort study at the University of Dundee Medical School.

**Table 1:** Structure of the mMERIT Incident Review Report, relationship between the 11 questions in mMERIT and the original 18 MERIT items and examples of expected standard for assessors. Examples of highly satisfactory content are provided at the end of each of the three sections

Incident Review Report	Original 18 MERIT Items	Guide for Assessors
Personal Learning	Factor 1: Personal Characteristics of QI	Examples of expected standard
1. What do you think were the contributing factors for the doctors involved in this incident?	• Quality of reflection on doctors practice • Sufficient details to delineate contributing factors	Demonstrates an understanding of the situation and can discuss the contributory factors within their incident and discusses in detail.
2. What could the doctors do to avoid similar problems in the future?	• Relevant new behaviours were proposed	Communicate with the team, patient, family Take a break, eat Find a more suitable environment to do task Consult a senior, Use protocols
3. What personal learning needs have you identified from this incident review?	• Doctor questioned their readiness to practice. • Multiple options for personal change were considered. • Contributing personal factors were identified	Characteristics – both technical and non-technical skills – readiness to practice i.e. lateness, attention to detail, memory Use of checklists, memory aids, asking for help
4. How will you meet these learning needs?	• Next steps towards personal change were identified.	Identifies specific ways to change
Personal Learning Score	Mark from 1 to 7: A score of 6 or 7 would include examples of: situational awareness, specific and timed learning objectives.	
Changes required to the system	Factor 2: System characteristics of QI	Examples
1. What do you think were the systems factors that contributed to this incident? Systems factors includes: the characteristics of the team and clinical setting where the incident took place, in addition to the organisation.	• Quality of reflection on the institution or wider health care system. • Current institutional practice or system was questioned • Contributing system factors were identified.	Culture – hierarchy structure, team work, communication between teams, different staff teams Environment e.g. noisy, lack of space to work cramped conditions, continually interrupted
2. What changes to the system might avoid similar problems in the future	• Relevant changes to the system were proposed • Next steps towards system change were identified	Use of multiprofessional handover, safety briefings, medicines reconciliation e.g. use of more than one source to confirm medications. Effective communication
3. What tests could be done to see if the changes might work?	• Multiple options for system change were considered	Testing any of the ideas above.
System Characteristics Score	Mark from 1 to 7: A score of 6 or 7 would include examples of changes to doctors and nurses working and small tests of change	
Why it is an important incident	Factor 3: Problem of Merit	Examples
1. What was the impact of this incident on the patient?	• Event was patient centred	Patient had an increased length of stay, patient had to undergo other investigations, patient developed infection, DVT, Investigations/theatre cancelled or delayed
2. How likely is it that similar incidents could affect other patients?	• Potential for event to affect other patients	Evidence of Impact of this incident on other patients
3. What is the worst that could happen to a patient because of an incident like this?	• Event could cause negative clinical impact • Overall problem of merit	Recognising the worst consequences from this incident e.g. The patient could have lost the wrong leg, required renal replacement, patient had to be admitted to HDU/ICU.
4. Event was evidence based in description	• Quality gap established from standards and guidelines (local or national)	Evidence of further reading, highlights local /national guidelines, relates other initiatives to incident, examples of good practice e.g. use of new folder for current admission.
Incident Importance Score	Mark from 1 to 7: A score of 6 or 7 would include examples of patient involvement and of negative impact on patient and public confidence in the NHS or on patient experience	
Overall Score	Mark from 1 to 7	
^*^Guide to scoring (1–7)	Description	
1–2	Concern	
3–5	Satisfactory	
6–7	Highly satisfactory	

The adapted mMERIT tool changed the context of the incident from reflecting upon an incident a junior doctor has encountered (MERIT) to giving the students an incident that had been reported by someone else but involved a junior doctor (mMERIT), We retained the three principals factors in MERIT but condensed the 18 items in MERIT into 11 questions for students to answer in their mMERIT reports (Table 1).

In each year of the study all final year students were required to complete an Incident Review during one of their two Foundation Year Assistantship blocks These are one month blocks that occur between September and May in each Academic Year. Students were assigned to groups of three to six and allocated an incident that had recently been reported on Datix, the online system that is used by NHS Tayside for incident reporting [8]. The incidents were chosen because they involved important care processes for Foundation Year doctors (e.g. prescribing and handover). Groups of students discussed the incident with a Senior Charge Nurse or Consultant who was familiar with the context where the incident took place and each student then submitted their reflective reports on the structured mMERIT form. A tutor provided written feedback with specific comments linked to text in each of the three mMERIT sections: personal learning, systems changes and incident importance (Table 1). Students were given a mark out of seven for each of the sections, an overall mark and general comments. mMERIT reports with concern in any of the three sections were returned to the student for resubmission.

### Year 1: 2011–2012

#### Student preparation

During the Final Year Induction week all students attended a two hour tutorial in groups of up to 40 after they had watched a dramatic reconstruction of a fatal adverse incident made by the World Health Organisation^3^. The tutorial was in Team Based Learning format [9]. In the first half of the tutorial students were asked to identify contributory factors for the incident and to discuss issues such as culture, hierarchy, team working and handover. In the second half of the tutorial students were introduced to the mMERIT structured report (Table 1). Following the tutorial students completed an online course called ‘Managing an Incident Review’ based on training given to staff within NHS Tayside [5].

#### Incident review

Senior Charge Nurses were recruited to mentor the students because they worked in the ward environment where the incidents took place and had reviewed the original incident reports to identify what should happen next. The students were expected to organise time with the mentor to discuss the incident.

The first 62 student reports were used to assess inter-rater reliability (described in detail under Methods Research Question 1). Subsequent reports were marked by a single assessor (VT or PD). However to ensure a consistency between markers, students’ reports that were marked as a concern were reviewed by another marker to ensure reliability.

All students were required to submit a report and include this in their Final Year Portfolio. Failure to submit a report was notified to the Medical School Office. Unsatisfactory reports were returned to the students for revision.

### Year 2: 2012–2013

In 2012–13 preparation of students was unchanged with the exception that the Application Test in the Team Based Learning tutorial was adapted so that students discussed and marked an anonymised student report from the previous year. The students were also asked to use the mMERIT template to record their reflection to each of the eleven questions. The goal was that this enhanced preparation would enable all Final Year students to submit a satisfactory report first time. Students were encouraged to include tests of change for service improvement. All reports were marked by VT or PD.

### Year 3: 2013–2014

No significant changes were made.

### Year 4: 2014–15

The Medical School made substantial changes to the curriculum for Preparation in Practice (Years 4 and 5). This meant that Final Year Induction was shortened to a single day, which was timetabled immediately after the students’ final online examination. The introduction to Incident Review had to be delivered to all Final Year students in a single one hour lecture and there was no time for students to watch the WHO Learning from Errors video [6] before the lecture so we included viewing of the video in the lecture.

We recruited three additional markers two Consultant Physicians (AC, JS) and the Medical School Lead for Behavioural and Social Science (EF). Management of allocation of students to groups, submission of student reports and resubmissions was taken over by the Medical School Undergraduate Office.

### Year 5: 2015–16

Students entering Final Year in 2015 had already undertaken a Significant Event Analysis (SEA) in primary care, which was introduced to the curriculum during Fourth Year in 2014–15. SEA was already in place in every general practice in NHS Tayside and students were enabled to identify an event for discussion and reflection at a practice team meeting. In addition students had a timetabled tutorial led by a GP for peer discussion of their SEAs. There was no assessment of the reflection on SEA.

We removed the WHO Learning from Errors video from the lecture on Incident Review in order to give students more time for marking and discussion of mMERIT reports.

### Research questions

1. What are the results for (overall, inter –rater and internal consistency) forms of reliability for assessment of the provided critical incident reports using the adapted mMERIT tool
2. Is there any relationship between assessment marks and the time in the academic year when students complete the assessment?
3. What explains variation in student performance?

#### Methods research question 1: mMERIT assessment reliability studies

##### Participants, materials and study process

The first 50 student reports received during 2011–2012 were assessed independently by three medical school staff assessors (VT, PD and Wendy Sayan). Each assessor had a background in, and responsibility for, Quality Improvement in medical school education. VT is a nurse and was a Specialist Nurse in Surgical High Dependency Unit before joining NHS Tayside’s Patient Safety team. PD is an Infectious Diseases Physician. Wendy Sayan is currently Head of Service, Child and Adolescent Mental Health in NHS Tayside and was a Patient Safety Manager in 2011–12. Each of the three assessors attended a meeting in order to familiarise themselves with the study’s eleven question mMERIT tool, which was used for all of the students’ assessments. The three assessors then independently marked three sets of four student reports in order to calibrate the mMERIT assessment before a final version was produced for testing of inter-rater reliability with a new set of 50 student reports.

Each of the 50 student reports were sent to each of the three Medical School assessors, who independently marked the report using the 11 question mMERIT tool. Student scores were entered into an excel spreadsheet which was then imported into GENOVA and its associated wrapper programme GS4 for analysis of the reliability of mMERIT using Generalizability G-Theory.

### Statistical analyses

Reliability analyses were conducted to test the reliability of mMERIT for its capacity to provide formative feedback to help steer students’ appropriate reflection on a provided clinical critical incident review.

Statistical analyses used Generalizability G-theory [10] to investigate the variance between the study facets (students, mMERIT questions and assessors) to test the reliability of mMERIT. Generalizability G-study and associated Decision D-studies, for different combinations of number of assessors, were investigated using urGENOVA and its associated statistical programme G-String I [11–13].

G-theory was selected for the study’s analysis in order to account for the multiple potential sources of error in the reliability calculations. Restricting the study’s analysis to classical test theory by calculation of Cronbach’s alpha on its own would have inflated mMERIT’s reported reliability by not accounting for error attributed to assessors [13]. The use of G-theory also allowed the mathematical manipulation of study results by the use of associated Decision D-studies [13]. Decision D-studies use the components of variance calculate from the original G-study to exploration of the most efficient number of assessors needed to achieve a level of reliability consistent with the proposed use of the assessment tool. The form of reliability investigated addresses the tool’s capacity to discriminate between students and account for the appropriate source of potential error from questions (internal consistency), or different assessors (inter-rater reliability) [13]. Appendix 1 gives explanations of the different forms of reliability analysed and gives copies of all of the formulae used in the calculations for the original G-Study and the associated D- Studies. The provision of the formulae used allows the reader to understand and, should they wish, replicate the study’s statistical methods and results. Results of these calculations using the study’s different components of statistical variance are provided later in the results section.

For calculating overall reliability, students were treated as the facet of differentiation and both raters and questions as facets of generalization. For calculating inter-rater reliability, students were treated as the facet of differentiation, questions as a fixed facet and assessors as the facet of generalization. For calculating internal consistency, students were treated as the facet of differentiation, raters as a fixed facet and mMERIT questions as the facet of generalization.

The interpretation of the reliability results, required of a measure, depend upon the assessment tool’s purpose. For high stakes assessment, such as in a pass/fail application high reliability (G > 0.8) would be required by a single stand-alone Workplace-Based Assessment (WPBA) tool [14]. However, for purposes of formative feedback to drive quality and/or personal improvement, or if as part of a suite of feedback on performance on which a high stakes overall assessment is made [14–16], lower levels of reliability of individual tools would suffice. For example, Objective Structured Clinical Examinations (OSCE), commonly used to inform summative decisions in other contexts, commonly report reliability levels of approximately G = 0.6 [17].

#### Methods research question 2: Gradient of student performance over the academic year

##### Participants, materials and study process

We used mMERIT marks from all student reports in 2011–12. The hypothesis was that there may be a gradient in marks across the Academic Year. A negative gradient could occur because students doing their incident review at the start of the year had better recall of the tutorial content. A positive gradient could occur because students doing their incident review at the end of the year would be in their second Foundation Year Assistantship block and would be more familiar with the system they were working in.

##### Statistics

We investigated the differences between student blocks (*n* = 35) for mean mMERIT scores (Q1–4). Levene’s test was used to check that there was no significant difference in the homogeneity of variance of data between the student blocks (n = 35) in order to check that the one way ANOVA test satisfied the homogeneity of variance assumption. One way ANOVA was then used to investigate the presence of any mean difference between the different student blocks’ section headings scores (mMERIT Q1–4). A Bonferroni correction was used to account for the multiple comparisons involved with alpha set at 0.001.

#### Methods research question 3: Explanation of variation in student performance.

Our ability to answer Research Question 3 was limited by the data available for analysis. During the calibration and reliability assessment of the first 50 students in 2011 we noted that a minority used the 3–4 questions in each of the three sections of mMERIT (Table 1) to structure their reflective reports. We therefore decided to focus on the impact of use of mMERIT Section Questions on the quality of student submissions. Our hypothesis was that marks would be higher for students who used Section Questions because it would be easier for them to see if they had answered all of the questions.

##### Participants, materials and study process

We used all student reports from 2011 to 12 and repeated the analysis with all reports from 2013 to 14.

##### Statistics

The outcomes for this analysis were the mean mMERIT scores in each of the three sections of the report (Q1 Personal learning, Q2 Systems change, Q3 Incident importance) and the overall global score (Q4). The analysis compared outcomes for students who had or had not used mMERIT Section Questions to help structure their submitted reflective reports. The number of Section Questions was four for Personal learning, three for Systems change and four for Incident importance (Table 1). Two cohorts of students were analysed 2011–2012 (n total = 153) and 2013–2014 (n total = 169). In the 2011–2012 cohort, 11 of the 153 students did not use the provided headings, and in the 2013–2014 cohort, 27 of the 169 students did not use the headings. Levene’s test was used to check that there was no significant difference in the homogeneity of variance for either year cohort between the two groups of students analysed to check if the one way ANOVA test satisfied the homogeneity of variance assumption. One way ANOVA was then used to investigate the presence of any mean difference (*P* < 0.05) between the two groups’ section headings scores (mMERIT Q1–4).

### Ethics

Our analyses used anonymous data from core student assessments, which did not require approval from the School of Medicine Research Ethics Committee. The Chair of the Committee provided a letter to confirm that this study did not require review by the Committee.

## Results

From September 2011 to May 2016 incident review has been a core activity for final year medical students at the University of Dundee. Each year students worked in 34 groups with 4–6 students per group. A total of 824 students have completed an incident review using 167 incidents from NHS Tayside’s incident reporting system. This has included incidents from 27 different ward areas across general medicine and surgery within two acute teaching hospitals within NHS Tayside.

### Research question 1: Inter-rater reliability

The overall reliability for the study’s three assessors marking the mMERIT eleven questions was very high and consistent with a level required for high stakes assessment G = 0.87 (the calculation is shown in Appendix 1).

The reliability pilot demonstrated mMERIT questions (*n* = 11) as having high overall reliability (G = 0.71–0.87) when based on results of between one and three assessors (Table 2). Inter-rater reliability results of (G = 0.56–0.79) was achieved between one and three assessors (Table 2). mMERIT showed high internal consistency (Cronbach’s alpha) (G = 0.95) (Table 2).

**Table 2:** Results of Decision D- Studies for Overall, Inter-rater and Internal Consistency reliabilities for different combination of observations

Overall Reliability		
Number of Questions	Number of Raters	G
11	1	0.71
11	2	0.82
11	3	0.87
Inter-Rater Reliability		
Number of Questions	Number of Raters	G
1	1	0.56
1	2	0.72
1	3	0.79
Internal Consistency		
Number of Questions	Number of Raters	G
11	1	0.95

(Calculations based on formulae given in Table 1)

### Research question 2: Gradient of student performance over the academic year

Our analysis did not reveal a trend in marks over time. Levene’s test showed that despite the number of students varying between different blocks (*n* = 19–27), there was no significant difference in population variances between blocks (*p* = 0.008–0.05 > 0.001 = alpha). Analysis by ANOVA was therefore appropriate and satisfied the homogeneity of variance assumption.

F (34,134) = 1.67–2.01, *P* > 0.001 in all cases.

### Research question 3: Impact of use of mMERIT section questions on the quality of student submissions

#### 2011–2012 student cohort

In year one of the study 11/153 (7.2%) of students used the Section Questions to construct their reflective reports. mMERIT mean scores and 95% confidence intervals for students who used Section Questions to structure their answers versus those who did not in Year 1 (2011–2012) are given in Table 3a.

**Table 3:** mMERIT scores by section comparing students who used section headings to structure their answers versus those who did not

a: Year 1 (2011–2012)					
MERIT		Used section headings (N = 11)		No section headings (*n* = 142)	
		Mean	95% CI	Mean	95% CI
Section 1	Personal learning	5.5	4.9–6.0	4.5	4.3–4.7
Section 2	Systems changes	5.3	4.7–5′8	4.4	4.2–4.6
Section 3	Incident importance	4.7	4.0–5.5	3.9	3.7–4.1
Overall Score		5.3	4.7–5.8	4.3	4.1–4.5
b: Year 3 (2013–2014)					
MERIT		Used section headings (N = 142)		No section headings (n = 27)	
		Mean	95% CI	Mean	95% CI
Section 1	Personal learning	5.7	5.6–5.8	4.4	3.9–4.9
Section 2	Systems changes	5.5	5.3–5.6	4.2	3.2–4.7
Section 3	Incident importance	5.4	5.3–5.6	3.8	3.3–4.3
Overall Score		5.5	5.4–5.7	4.0	3.6–4.5

Levene’s test showed that despite the number of students varying between those not using section headings (*n* = 11) and using section headings (*n* = 142), there was no significant difference in population variances between groups for mMERIT section break questions 1–4 (*p* = 0.06–0.74 > 0.05 = alpha). Analysis by ANOVA was therefore appropriate and satisfied the homogeneity of variance assumption.

Comparison of group means using one way ANOVA demonstrated a significant difference between students groups F[1151] = 4.97–7.66, *p* < 0.05 for all mMERIT questions 1–4.

#### 2013–2014 student cohort

In year three of the study, 142/169 (84%) of students used the section headings to construct their reflective reports. mMERIT mean scores and 95% confidence intervals for students who used section headings to structure their answers versus those who did not in Year 3 (2013–2014) are given in Table 3b.

mMERIT scores by section comparing students who used section headings to structure their answers versus those who did not in Year 3 (2013–2014) are given in Table 3b.

Levene’s test was again used with this year’s cohort of students to explore the homogeneity of variance between those not using (*n* = 27) and those using (*n* = 142) section headings. There was no significant difference in population variances between the two student groups for mMERIT section break questions 2 (*p* = 0.12 > 0.05 = alpha) and global rating mMERIT question 4 (*p* = 0.06 > 0.05 = alpha). Analysis by ANOVA was therefore appropriate for question 2 and 4, and satisfied the homogeneity of variance assumption. There was, however, a significant difference in population variances between the two student groups for mMERIT section break questions 1 (*p* < 0.001) and question 3 (*p* < 0.05), indicating that the ANOVA test was too sensitive to assess differences in group means for mMERIT questions 1 and 3, given the two groups of students’ unequal sample sizes.

Comparison of group means using one way ANOVA demonstrated a significant difference between students groups who either used or did not use section break headings, for mMERIT questions 2 and nMERIT global rating question 4, (F = [1167] = 43.87; 72.96, p < 0.001) respectively.

#### Longitudinal results and sustainability

The number of students in Final Year has increased by 22% from 153 in Year 1 to 186 in Year 5 (Table 4). Each year all students have completed the incident review. Reports marked as concern were returned for resubmission and all students have had a satisfactory mMERIT report in their portfolios by the end of Final Year.

**Table 4:** Number of students per year and the number with mMERIT reports marked concern or highly satisfactory

Year of study	2011–12	2012–13	2013–14	2014–15	2015–16
N students	153	159	169	157	186
Concern	21	9	5	11	10
% (95% CI)	14% (8–19%)	6% (2–9%)	3% (1–6%)	7% (3–11%)	5% (2–9%)
Highly satisfactory	26	54	70	38	59
% (95% CI)	17% (11–23%)	34% (27–41%)	41% (34–49%)	24% (18–31%)	32% (25–38%)

**Fig. 1:**
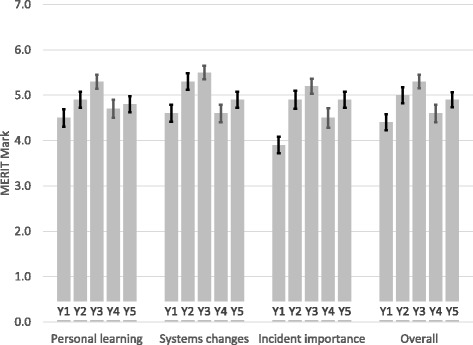
mean (95%CI) of marks for each section and overall by study year from 2011 to 12 (Y1) to 2015–16 (Y5) and summary of key changes to preparation of students. Key changes to preparation of students. Y2 (2012–13): clearer instructions to use question headings to structure reflective reports in each of the three sections of the report. Y4 (2014–15): reduction in time available for preparation of students and in format from tutorial to lecture theatre

In comparison with 2011–12 there was an increase in marks across all sections of mMERIT in subsequent years (Fig. 1). In 2011–12 21 (14%) of 153 mMERIT reports were graded as concern compared with 3–7% of reports in subsequent years (Table 4). In 2011–12 the marks for Incident Importance were lower than for the other two sections but this did not occur in subsequent years (Fig. 1). Pooled results of 671 mMERIT reports for the four years from 2012 showed that, in comparison with the other two sections, the Systems Improvement section was more likely to be marked Highly Satisfactory. Systems Improvement was rated highly satisfactory in 41% of reports in comparison with 30% for the other two sections (Table 5) and was more likely to be rated highly satisfactory in each of the four years (data not shown). This means that only a third of our students are achieving highly satisfactory grades on mMERIT and that the main areas of weakness are in personal development planning (Personal learning) and in demonstrating empathy for patients, families and staff (Incident importance).

**Table 5:** Grading of mMERIT reports by section for 671 students in the four years from 2012 to 16

Section of report	Concern	Satisfactory	Highly Satisfactory
*N* = 671 students	% (95% CI)	% (95% CI)	% (95% CI)
Personal learning	21	447	203
	3% (2–4%)	67% (63–70%)	30% (27–34%)
Systems changes	12	386	273
	2% (1–3%)	58% (54–61%)	41% (37–44%)
Incident importance	28	442	201
	4% (3–6%)	66% (62–70%)	30% (27–33%)
Chi-square	26.63, *p* = 0.00002 with 2 degrees of freedom		

There was a decrease in marks in 2014–15 (Fig. 1). We attributed this to changes in the Final Year Induction Week, which reduced the time for preparation of students from two to one hour and changed the format from tutorials with 40 students to a lecture delivered to all students. Marks improved in 2015–16 after we changed the content of the introductory lecture to remove the WHO Learning from Errors video and focus entirely on marking of mMERIT reports from the previous year.

In the four years from September 2012 to May 2016 we graded 221 of 671 reports as highly satisfactory: 33% (95% CI 29–36%). This means that only a third of our Final Year students are making specific, timed learning objectives, demonstrating awareness of tests of change for systems improvement and patient involvement in assessing the importance of incidents (Table 1).

## Discussion

We have shown that mMERIT has high reliability with one rater. There was no evidence of any gradient of student performance with mMERIT across the Final Year. Student performance was enhanced by reminding them to answer all eleven questions in mMERIT. mMERIT can be used by students as part of a suite of feedback to help supplement their self-assessment on their leaning needs and develop insightful practice to drive their development of quality, safety and person centred professional practice [14–16, 18].We were able to sustain incident review as a core component in Final Year over five years despite a 22% increase in student numbers and reduction in curriculum time for classroom preparation of students.

Incident review addresses the need for workplace based learning and use of real life examples of mistakes identified by previous studies of education about patient safety in medical schools [1–4, 19, 20]. Using mMERIT for assessment of incident review addresses each of the seven key challenges to patient safety education that were identified in a qualitative study with faculty from Schools of Medicine, Nursing and Pharmacy [3].

1. Clinical safety areas: we have involved Senior Charge Nurses and Consultants from the setting where incident took place and was reported by a member of the clinical team.
2. Priority setting: incident review is a required element of the Final Year Portfolio and co-ordination is managed by staff in the Medical School Undergraduate Office.
3. Culture of the clinical practice setting: students are asked to review and reflect on an incident that the clinical team had already identified as important
4. Formal vs informal: incident review is integrated into both clinical teaching and assessment in Final Year
5. Faculty preparation: mMERIT enables standardised preparation of faculty for assessment of reflective reports.
6. Authenticity: the incident review is workplace based and the mentor is a senior member of the clinical team
7. Academic-practice gap: our current Faculty is multi-professional (nurse, psychologist, doctors) and includes a mix of clinical and academic staff.

A systematic review of literature published from 2009 to May 2014 identified eleven patient safety education interventions for medical students that included an evaluation [2]. Although six (55%) of courses included root cause or systems based analysis only one included error disclosure and none included incident reporting (methods, barriers) [2]. The median number of students per course was 120 (IQR 109–151) and only three (27%) included data from more than one year. Only two courses included any assessment of students and these were both limited to self-reported measure [2].

In addition to core, workplace based teaching on incident review we have introduced optional courses on healthcare improvement into second, third and final year. Examples of successful student improvement projects can be found on the IHI Open School website [21] and in BMJ Quality Improvement Reports [22–24]. The contribution of final year medical students to the Patient Safety Network was recognised in a report to NHS Tayside Health Board in 2014 [25]. This provides evidence of progress towards three of the five core elements of the Exemplary Care and Learning Sites model: student/trainee engagement in the improvement of care; leaders knowing, valuing and practicing improvement and health professionals competently engaging both in care improvement and teaching about care improvement [26]. However we need to build capacity because only 30–40 of our students are actively engaged in improvement in any academic year. We also need to develop new opportunities for students to work with patient and families on informing process changes [26].

### Limitations

We have been unable to assess impact on practice in Foundation Year (FY) because less than 50% of our graduates work in NHS Tayside. Lack of information about the impact of patient safety education on behaviour in practice or on outcomes for patients or systems was identified as a weakness of all included studies in a recent systematic review [2]. We considered trying to look at differences in skills and attitudes between young doctors who graduated from Dundee compared with other Medical Schools. However, the Postgraduate Deanery in NHS Tayside decided to introduce training and assessment of incident review for all doctors early in their first year of training, which would make meaningful comparison difficult. We introduced this training in 2011 and it was associated with a 17-fold increase in reporting of incidents by Foundation Year doctors in NHS Tayside [27].

The Medical School manages feedback on core curriculum and it has not been possible to include specific questions about Incident Review within feedback about the Foundation Assistantship Blocks. We need to be more assertive by arguing that this implies that the Medical School regards patient safety and the incident review as being peripheral to the preparation and assessment of future doctors.

Only a third of our students are achieving highly satisfactory grades on mMERIT. We believe that improvement will require changes to the curriculum. We have already introduced new classroom teaching on patient safety, human factors and inter-professional teamwork from Year 1 since 2013. However, we also need to develop workplace based opportunities for learning about patient experience, systems thinking and acquiring the habits of an improver [19, 28, 29]. Our results show that most of our final year students are not very good at planning their personal development or reflecting on person centred care, which are key skills for practicing in and leading in complex systems [19].

## Conclusions

We have shown that mMERIT has high reliability with one rater. Incident review addresses the need for workplace based learning and use of real life examples of mistakes identified by previous studies of education about patient safety in medical schools [1–4]. Our experience shows that mMERIT provides a structured, sustainable method for preparing students for incident review in practice that requires little curriculum time. We recommend that other medical schools consider introducing incident review into their curricula.

## Appendix

Formulae used for calculation of each form of reliability and an example of application to calculation of overall reliability from study results.

### D-study formulae used for calculations and the results for overall, inter-rater and internal consistency forms of reliabilities

Overall Reliability: Accounts for error generalized across case scenarios, raters and questions and gives the overall test reliability for the given combination of observations.

*Formula*: σ2 (student)/ (σ2 (student) + σ2 (student*rater/ n raters) + σ2 (student*question/ n questions) + σ2(student*rater*question)/(n raters*questions) =.

Inter-Rater Reliability: Accounts for error generalized across different raters and gives the inter-rater correlation for another rater (based on correlation with mean of given number of existing raters’ scores).

*Formula*: (σ2 (subject) + σ2 (subject*question) / (σ2(subject) + σ2(subject*question) + σ2 (subject*rater/ n raters) + σ2 (subject*rater*question)/n rater) =.

Internal Consistency: Accounts for error generalized across different questions in an assessment inventory. It gives the internal consistency (across questions) for one rater.

*Formula*: (σ2 (subject) + σ2 (subject*rater) / (σ2 (subject) + σ2(subject*rater) + σ2 (subject*question/ n questions) + σ2 (subject*rater*question)/n questions) =

### Study facets and estimated variance components for 50 students, 3 raters and 11 questions in mMERIT

Study facets

Student: Facet of differentiation, *n* = 50.

Rater: Facet of Generalisation, *n* = 3.

Question: Facet of Generalisation, *n* = 11.

**Table:**

Effect	Degrees of Freedom	Mean Squares	Estimated variance
Student	49	38.59	1.02
Rater	2	90.73	0.15
Question	10	7.72	0.03
Student*Rater	98	4.45	0.35
Student*Question	490	1.14	0.18
Rater*Question	20	2.28	0.03
Student*Rater*Question	980	0.60	0.60

#### Calculation of G-coefficient (overall reliability):

σ2 (student)/(σ2 (student) + σ2 (student*rater/ 3) + σ2 (student*question/ 11) + σ2.(student*rater*question)/33 = 0.87

## Abbreviations

MERIT: Mayo Evaluation of Reflection on Improvement Tool
mMERIT: modified Mayo Evaluation of Reflection on Improvement Tool
NHS: National Health Service
SEA: Significant Event Analysis
WHO: World Health Organisation
